# Hyperpigmentation after surgery for a deep dermal burn of the dorsum of the hand: partial-thickness debridement followed by medium split-thickness skin grafting *vs* full-thickness debridement followed by thick split-thickness skin grafting

**DOI:** 10.1186/s41038-016-0039-7

**Published:** 2016-05-05

**Authors:** Yoshitaka Kubota, Nobuyuki Mitsukawa, Kumiko Chuma, Shinsuke Akita, Yoshitaro Sasahara, Naoaki Rikihisa, Kaneshige Satoh

**Affiliations:** 1 Department of Plastic Surgery, Chiba University, 1-8-1, Inohana, Chuo-ku, Chiba, Chiba 260-8677 Japan; 2 Department of Plastic Surgery, Tokyo Rosai Hospital, 4-13-21, Omoriminami, Ota-ku, Tokyo 143-0013 Japan; 3 Department of Plastic Surgery, Chiba Rosai Hospital, 2-16, Tatsumidaihigashi, Ichihara, Chiba 290-0003 Japan

**Keywords:** Hand burn, Hyperpigmentation, Skin graft

## Abstract

**Background:**

Early excision and skin grafting are commonly used to treat deep dermal burns (DDBs) of the dorsum of the hand. Partial-thickness debridement (PTD) is one of the most commonly used procedures for the excision of burned tissue of the dorsum of the hand. In contrast, full-thickness debridement (FTD) has also been reported. However, it is unclear whether PTD or FTD is better.

**Methods:**

In this hospital-based retrospective study, we compared the outcomes of PTD followed by a medium split-thickness skin graft (STSG) with FTD followed by a thick STSG to treat a DDB of the dorsum of the hand in Japanese patients. To evaluate postoperative pigmentation of the skin graft, quantitative analyses were performed using the red, green, and blue (RGB) and the hue, saturation, and brightness (HSB) color spaces. We have organized the manuscript in a manner compliant with the Strengthening the Reporting of Observational Studies in Epidemiology (STROBE) statement.

**Results:**

Data from 11 patients were analyzed. Six hands (five patients) received grafts in the PTD group and eight hands (six patients) received grafts in the FTD group. Graft take was significantly better in the FTD group (median 98 %, interquartile range 95–99) than in the PTD group (median 90 %, interquartile range 85–90) (*P* < 0.01). Quantitative skin color analyses in both the RGB and HSB color spaces showed that postoperative grafted skin was significantly darker than the adjacent control area in the PTD group, but not in the FTD group.

**Conclusions:**

There is a possibility that FTD followed by a thick STSG is an option that can reduce the risk of hyperpigmentation after surgery for DDB of the dorsum of the hand in Japanese patients. Further investigation is needed to clarify whether the FTD or the thick STSG or both are the factor for the control of hyperpigmentation.

## Background

Early excision and skin grafting are commonly used to treat deep dermal burns (DDBs) of the dorsum of the hand [1, 2]. However, postoperative pigmentation of grafted skin is a major problem, especially in colored people [3–6]. Because the hand is one of the most frequently exposed regions of the human body in everyday life, its appearance is strongly associated with one’s body image, which can affect the quality of life [7, 8]. Asian people are more prone to develop pigmentation than Caucasians because of differences in the physiological properties of their skin, especially in terms of melanocyte function and melanosome regulation [9–12].

In addition to race, differences in operative procedures can also affect postoperative pigmentation [6]. To optimize skin graft take, adequate debridement is essential [13]. With regard to the depth of debridement, partial-thickness debridement (PTD) (i.e., tangential excision, using punctiform hemorrhage as an indicator of the required depth of debridement) is one of the most commonly used procedures for excision of burned tissue of the dorsum of the hand [14]. In contrast, there have been reports of full-thickness debridement (FTD) performed using a tourniquet while exposing the pearly yellowish-white underlying tissue of the dermis and using it as an indicator of the required depth of debridement [15]. However, for deep dermal burns of the dorsum of the hand, it is unclear whether PTD or FTD is better, especially in terms of esthetic outcomes.

Our primary objective in this study was to compare the risks and benefits in Japanese patients, especially in terms of esthetic outcomes, of the two methods of surgery for deep dermal burns of the dorsum of the hand: (1) PTD followed by a medium split-thickness skin graft (STSG) and (2) FTD followed by a thick STSG.

## Methods

### Study design, setting, and ethics

We performed a hospital-based, retrospective cohort study comprised of Japanese patients who had received either a PTD (PTD group) or a FTD (FTD group). The study protocol was approved by the Ethical Committee of Chiba University (approval number 1673). Written consent was not required because patient records were anonymized and were de-identified prior to analysis. All studies were performed according to the guidelines of the Declaration of Helsinki. We have organized the manuscript in a manner compliant with the Strengthening the Reporting of Observational Studies in Epidemiology (STROBE) statement.

### Patient selection and study protocol

Patients aged 15 years or older who underwent skin graft surgery for a deep dermal burn of the dorsum of the hand at Chiba University Hospital in Japan and related hospitals in Japan between December 2007 and September 2010 were identified retrospectively from the operative register. We performed FTD followed by a thick STSG harvested from the dorsum of the trunk from May 2009. In the latter 17 months of the study period (from May 2009 to September 2010), all patients who underwent surgery for deep dermal burns of the hand were treated by FTD followed by a thick STSG. As a historical control, in the preceding 17 months of the study period (between December 2007 and April 2009), all patients who underwent surgery for a deep dermal burn of the hand were treated by PTD followed by a medium STSG. Patients judged by a senior plastic surgeon to have third-degree burns of the dorsum of the hand, based on preoperative photographs, were excluded.

Medical records were analyzed retrospectively for data containing the following information: age, gender, Fitzpatrick skin type, cause of burn, percentage of total body surface area (%TBSA) occupied by the burn area, %TBSA excised, %TBSA of the skin harvested, duration of tourniquet application, harvest site and thickness of skin graft, blood loss during surgery, amount of blood transfused during surgery, days of hand fixation after skin graft surgery, and percentage of graft take 7 days after surgery [16]. We also investigated the incidence of postoperative epidermal cysts in the dorsum of the hand, occurrence of hypertrophic scars and scar contracture of the hand, days required for healing of the donor site, modified Vancouver Scar Scale of the donor site (Table 1), and hyperpigmentation of grafted hands 12 months after surgery [17].

**Table 1:** Modified Vancouver Scar Scale

Score	Vascularity	Pliability	Height	Pigmentation
0	Normal	Normal	Flat	Normal
1	Pink	Supple	<2 mm	Hypopigmentation
2	Red	Yielding	2–4 mm	Mixed
3	Purple	Firm	>4 mm	Hyperpigmentation
4		Banding		

### Burn surgery

**Fig. 1:**
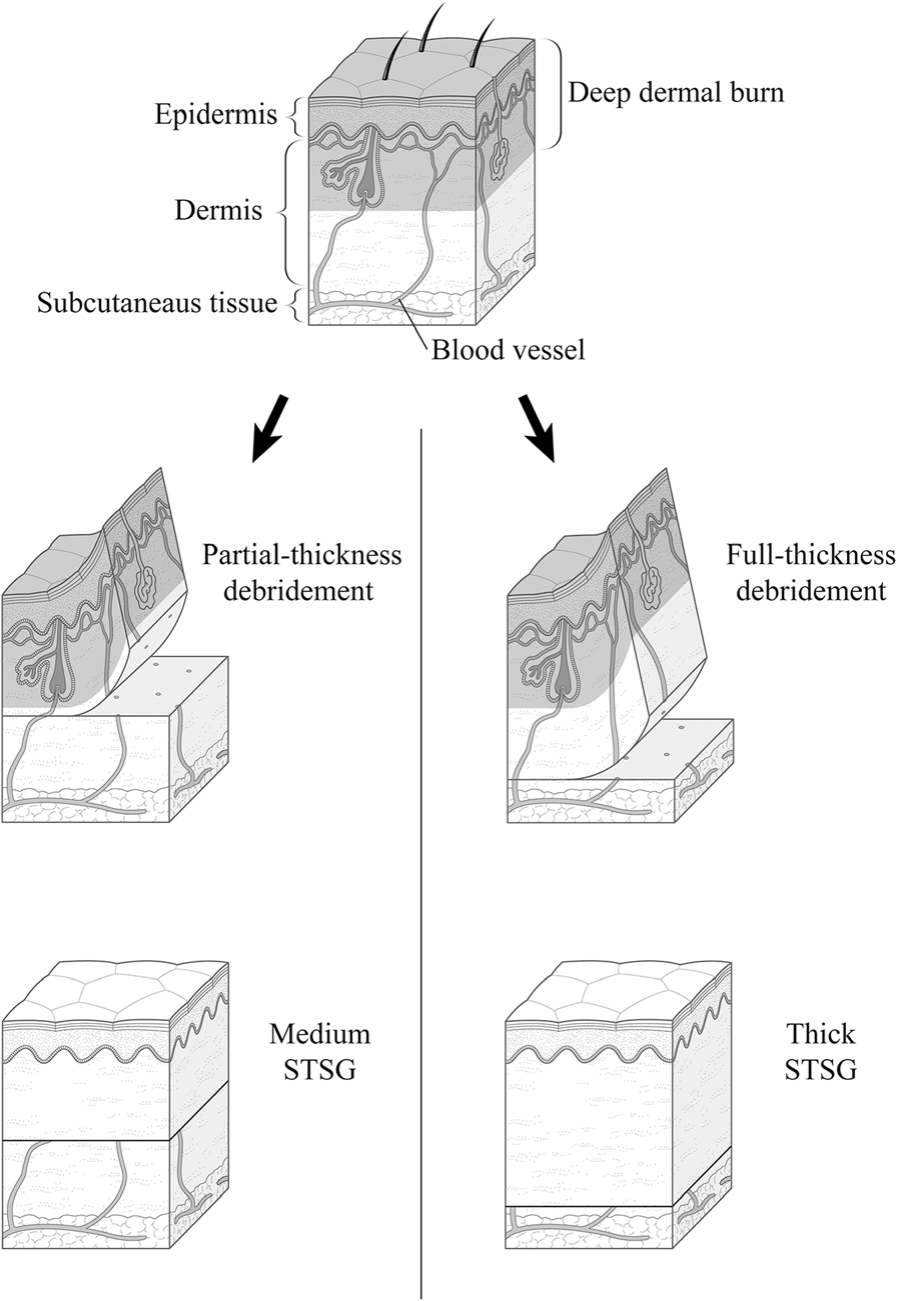
A schematic of the comparison made in this study. The *left column* shows a diagram of partial-thickness debridement (PTD), representative of the group in which tangential excision was performed to preserve the dermis of the hand as much as possible. After the tangential excision, a medium split-thickness skin graft (*STSG*) was applied to compensate for the loss of the dermis. The *right column* shows a diagram of full-thickness debridement (FTD), representative of the FTD group in which that procedure was performed. After the debridement, a thick *STSG* was applied to reconstruct the dermis of the hand

In the PTD group, tangential excision of the burn wound of the dorsum of the hand was performed with a dermatome knife without a tourniquet (Fig. 1). The burn wound was excised until punctate hemorrhage was observed. Using the Padgett-Hood dermatome, we harvested the STSG with a thickness of 12–13/1000 in from the lateral thigh or abdomen. The unmeshed skin graft was sutured to the dorsum of the hand. Using a no. 11 blade scalpel, we made small slits for drainage at intervals of 10–15 mm. The hand was fixed with a bulky dressing and a volar splint and was kept raised. The first dressing change after the operation was done 5–7 days postoperatively. Active range of motion exercises were started 10–16 days postoperatively. A volar splint was applied during the night for 1 month postoperatively.

**Fig. 2:**
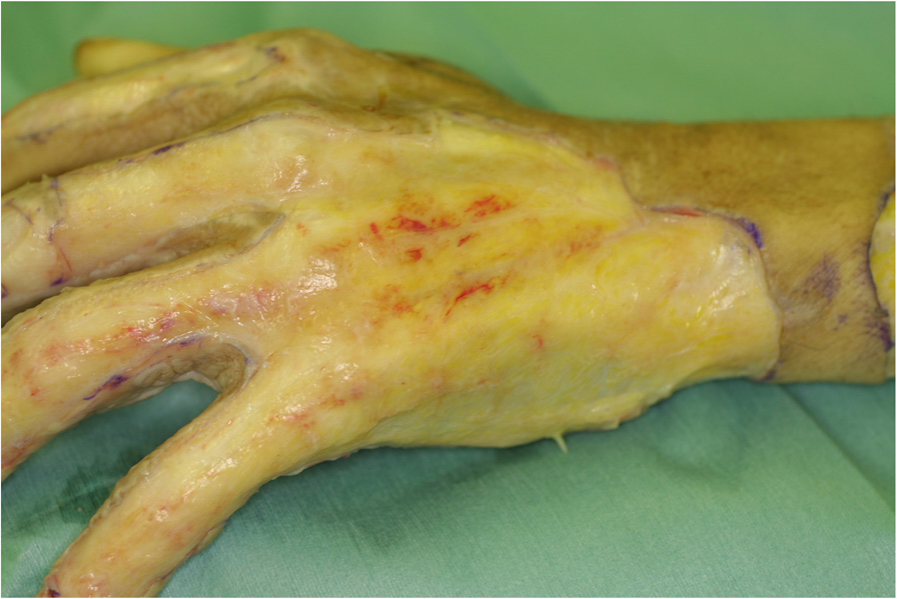
A photograph of the dorsum of a hand from a patient in the full-thickness debridement group who underwent debridement and tourniquet application. The pearly yellowish-white appearance of the tissue under the dermis is an indicator of the required depth for debridement. Note that the paratenon and subcutaneous veins are preserved

In the FTD group, the excision of the burn wound of the dorsum of the hand was performed with the use of a tourniquet inflated to 250 mmHg. We excised the burn wound until we exposed the pearly yellowish-white tissue underneath the dermis (Figs. 1 and 2). Careful attention was given to preserve the subcutaneous veins and the paratenon. After completion of the debridement, the tourniquet was deflated. The patient was changed to a lateral position or a prone position for skin graft harvesting. Using the Padgett-Hood dermatome, we harvested a STSG with a thickness of 25–30/1000 in from the posterior or lateral trunk. The patient was placed in a supine position, and the harvested skin was grafted to the dorsum of the hand. The hand was fixed in a grasping position around a gauze ball. The first dressing change was on the first postoperative day, and daily drainage of any blood under the skin graft was via a small postoperative incision. Fixation of the hand was removed 5–7 days after the operation, and active motion exercise was started. For a month postoperatively, a volar splint was applied during the night.

### Materials to evaluate esthetic quality of the hand

**Fig. 3:**
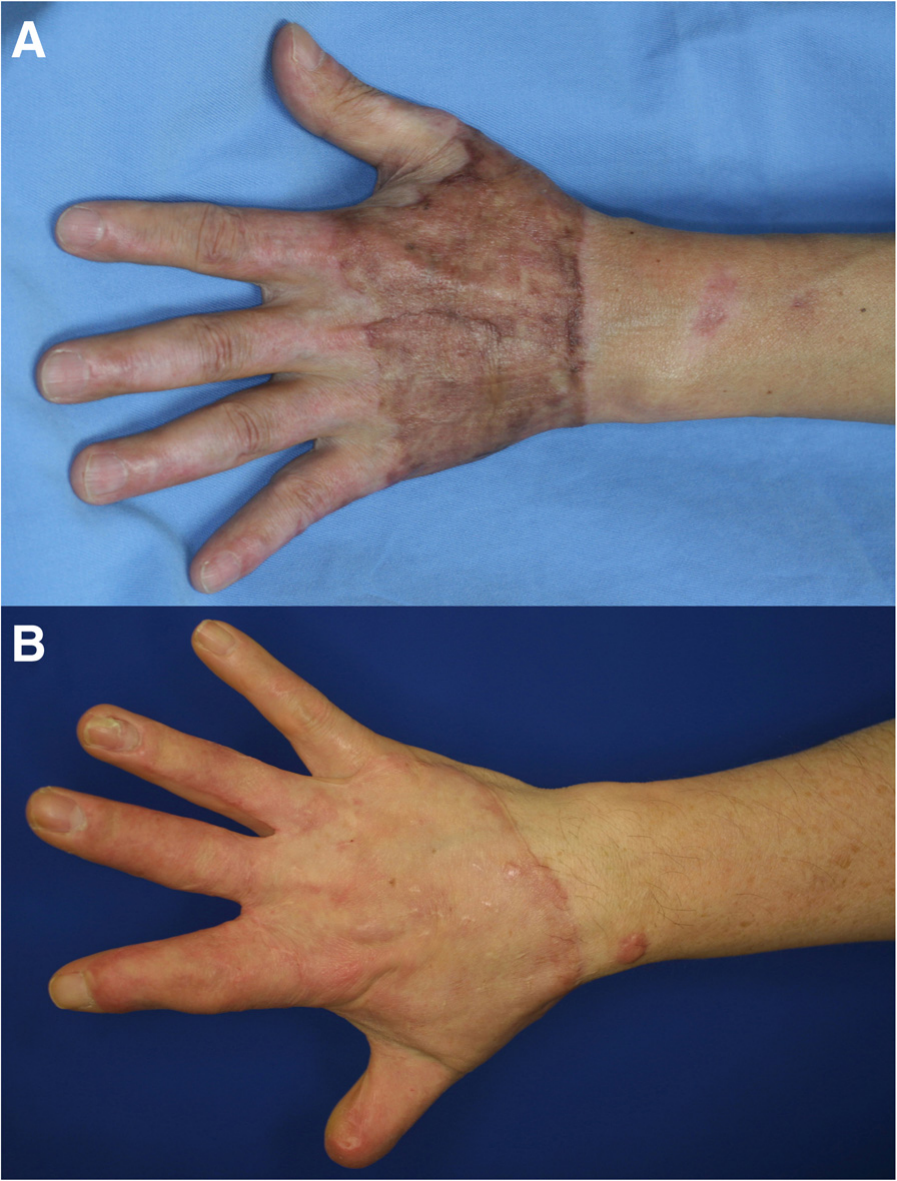
Photographs taken 12 months postoperatively. **a** The skin graft in the partial-thickness debridement group shows hyperpigmentation. **b** The skin graft in the full-thickness debridement group shows little pigmentation. Deformity of the thumb and index finger in this patient is due to previous trauma

**Fig. 4:**
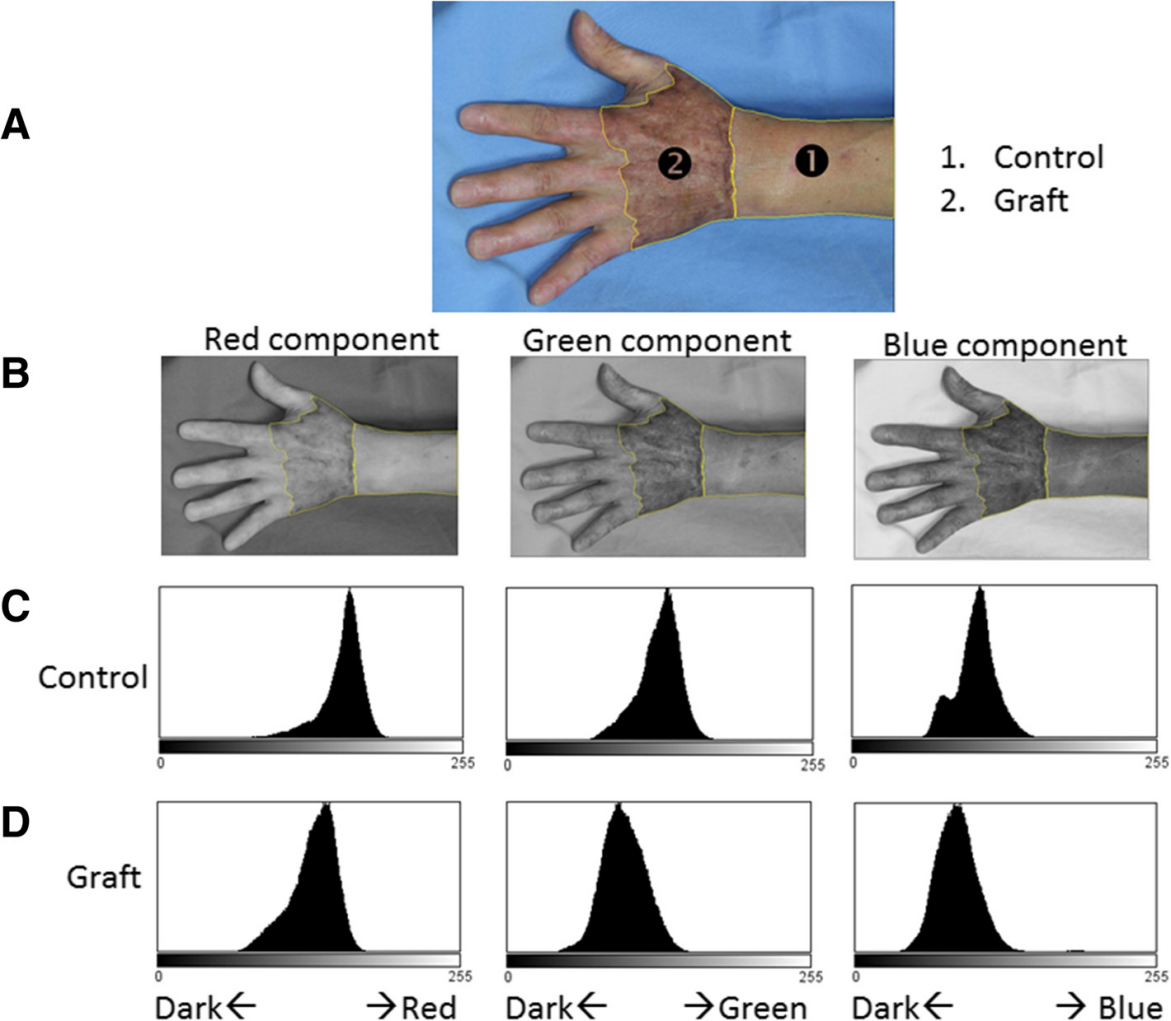
Two-dimensional (2D) quantitative analyses of skin color in the red, green, and blue (RGB) color space. **a** Skin graft area and control area were defined using the polygon selection tool. **b** Images from *left* to *right* split into red, green, and blue components, respectively. **c** Row of 2D histograms of each of the color components (control). **d** Row shows all three components and the distribution of the graft area is located on the *left side* (*darker side*) compared with the control

**Fig. 5:**
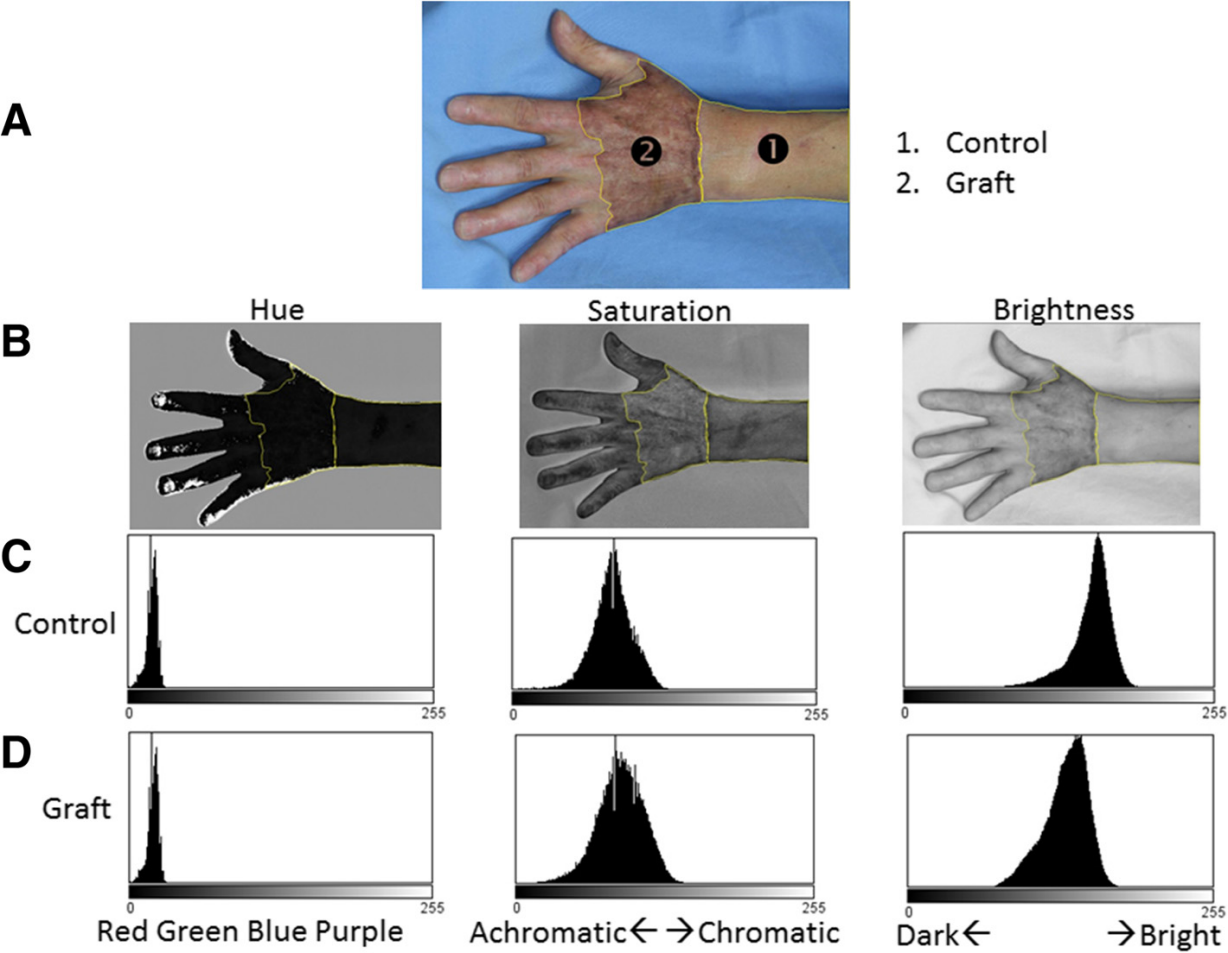
Two-dimensional (2D) quantitative analyses of skin color in the hue, saturation, and brightness (HSB) color space. **a** Skin graft area and control area. **b** Images from *left* to *right* in row split into hue, saturation, and brightness components. **c** 2D histograms for the control. **d** The histogram for the brightness component of the graft is left-sided compared with the control, indicating that the graft is darker than the control

Esthetic outcomes were compared with color photographs taken in an outpatient room of the Department of Plastic Surgery 12 months after the operation (Fig. 3). Color photographs were taken at a distance of 1.0–1.5 m from the patient’s hand, which was placed in front of a wall that provided a blue background. Equipment and conditions used for taking photographs were as follows: digital single-lens reflex camera, Canon EOS D30 or Canon EOS Kiss Digital X; lens, Canon EF 50 mm f/2.5 compact macro lens; photoflash, Canon MR-14EX macro ring lite flash; aperture value F2.5 or F5.6, exposure time 1/60 s, ISO 100 or 400; and image size of 2160 × 1440 square pixels with a resolution of 180 × 180 dpi, with 24 bits per pixel in the standard red, green, and blue (RGB) color space. Imaging analyses were conducted using ImageJ software (version 1.47; National Institutes of Health, Bethesda, MD) and ImageJ plugin software: Color Inspector three-dimensional (3D) (version 2.0; Barthel, KU; Berlin, Germany). The skin-grafted area was selected with a polygon selection tool. The adjacent wrist and forearm area was used as a control (Figs. 4 and 5).

### Quantitative analyses in the RGB color space

In the red, green, and blue (RGB) color space, the color of each pixel was defined by a combination of levels of three color components as follows: red intensity, green intensity, and blue intensity [18]. RGB 3D histograms, in a three-axes rectangular Cartesian coordinate system, were obtained using Color Inspector 3D software [19].

Levels of each of the three color components (red, green, and blue) were expressed on an 8-bit scale (256 integer value levels from 0 to 255). The meanings of the values are as follows: red intensity (0–255; 0, black; 255, red); green intensity (0–255; 0, black; 255, green); and blue intensity (0–255; 0, black; 255, blue).

Histograms and the weighted mean intensity values of each of the color components (red, green, and blue) were obtained (Fig. 4). Differences (Δ) in the color intensity of each color component between the skin graft area and the control area were defined as follows: Δred intensity = mean red value of skin graft − mean red value of control area; Δgreen intensity = mean green value of skin graft − mean green value of control area; Δblue intensity = mean blue value of skin graft − mean blue value of control area.

### Quantitative analyses in the HSB color space

In the hue, saturation, and brightness (HSB) color space, the color of each pixel was defined as a combination of three color components: hue, saturation, and brightness [20]. HSB 3D histograms in a cylindrical coordinates system and two-dimensional (2D) histograms were obtained in the same way as those in the RGB color space (Fig. 5).

**Fig. 6:**
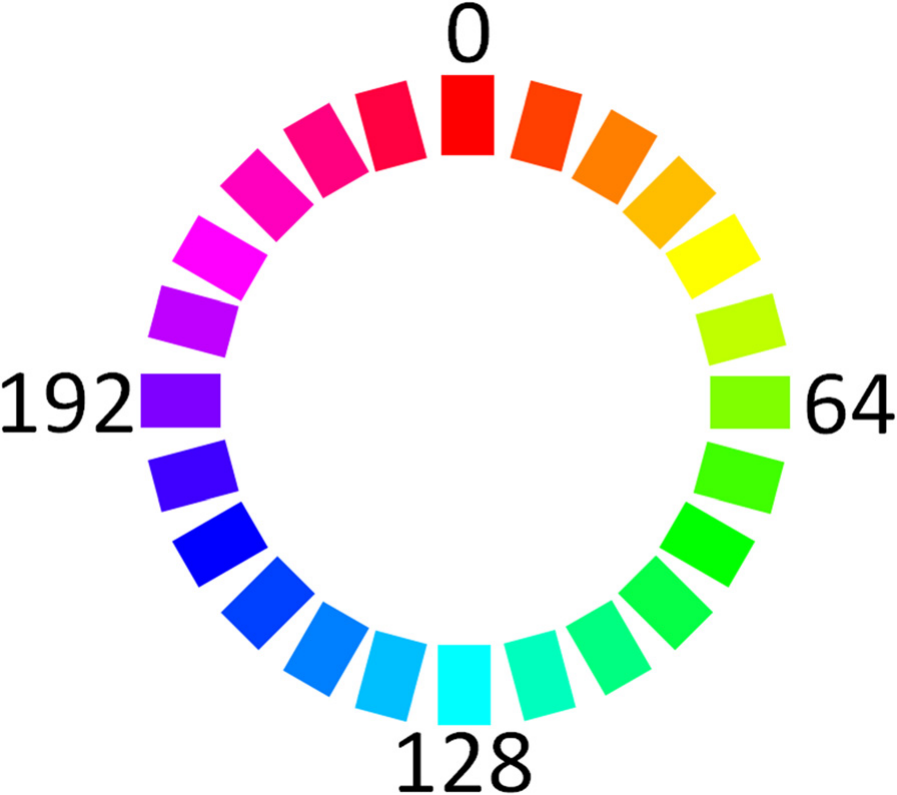
Correspondence between the hue component in the hue, saturation, and brightness (HSB) color space and the 8-bit scale

Values of color components in the HSB color space were defined on an 8-bit scale (256 integer value levels from 0 to 255). The meanings of the values are as follows: hue (0–255; 0, red; 64, yellow-green; 128, light blue; 192, purple; 255, return to red) (Fig. 6); saturation (0–255; 0, colorless; 255, chromatic); brightness (0–255; 0, dark; 255, bright). In terms of each color component, differences between the skin graft and the control area were defined as follows (Fig. 5): Δhue = mean hue value of skin graft − mean hue value of control area; Δsaturation = mean saturation value of skin graft − mean saturation value of control area; Δbrightness = mean brightness value of skin graft − mean brightness value of control area.

### Statistical analysis

Continuous variables are presented as median (interquartile range). Continuous variables were compared with Mann-Whitney *U* test. Categorical variables were compared with Fisher’s exact probability test. All *P* values quoted are two-tailed. *P* values less than 0.05 were considered to indicate statistical significance. Statistical analyses were conducted using SPSS software (version 13.0J; SPSS, Chicago, IL).

## Results

### Patient characteristics

A total of 11 patients met the criteria for the study (Table 2). Five patients received grafts in the PTD group and six patients received grafts in the FTD group.

**Table 2:** Patient characteristics

Characteristic	Debridement group		*P* value
	Partial-thickness (*n* = 5)	Full-thickness (*n* = 6)	
Age, years	52 (27–69)	67 (42–80)	0.43
Gender, *n*			1.00
Female	0	1	
Male	5	5	
Fitzpatrick skin type, *n*			0.79
Type II	2	3	
Type III	2	3	
Type IV	1	0	
Cause of burn, *n*			0.18
Flame	3	6	
Explosion	1	0	
Melted metal	1	0	
Burn area, %TBSA	14 (10–41)	17 (11–24)	0.66
Inhalation injury, *n*	4	2	0.57

Continuous variables are expressed as median (interquartile range)

*n* number of patients, *TBSA* total body surface area

There were no significant differences between the PTD group and the FTD group for age, gender, Fitzpatrick skin type, and cause of burn. There were no significant differences in burn area between the PTD group and the FTD [14 (10 − 41) vs 17 (11 − 24)%TBSA; *P* < 0.66].

### Extent of burn surgery

Six hands received grafts in the PTD group, whereas eight hands received grafts in the FTD group (Table 3). There was no significant difference in the mean number of days from burn injury to surgery between the PTD group and the FTD group [13 (8 − 24) vs 12 (8 − 19) days; *P* = 0.79]. No patient in either group underwent surgery of the palmar side of the hand. There was no significant difference in excised body surface area [4.0 (2.5 − 6.0) vs 4.0 (3.5 − 6.0)%; *P* = 0.50] and harvested body surface area [3.0 (2.0 − 4.0) vs 3.0 (3.0 − 6.0); *P* = 0.42] between the two groups. A tourniquet was not used in the PTD group, but was used for all patients in the FTD group [duration of tourniquet use, 68 (55 − 98) min]. With regard to the invasiveness of surgery, there was no significant difference in blood loss [275 (113 − 4617) vs 495 (366 − 2206) g; *P* = 0.54] or blood loss per %excision [275 (28 − 769) vs 100 (73 − 428) g/%TBSA; *P* = 0.93]. There were no significant differences in usage of either crystalloid or blood transfusion. In the PTD group, four thighs and two abdomens were used as skin graft donor sites, whereas in all patients in the FTD group, the dorsum of the trunk was used as a skin graft donor site (*P* < 0.01).

**Table 3:** Characteristics of burn surgery

Characteristic	Debridement group		*P* value
	Partial-thickness (*n* = 5)	Full-thickness (*n* = 6)	
Hands operated on, *n*	6	8	−
Time from burn to surgery, days	13 (8–24)	12 (8–19)	0.79
Preoperative hemoglobin, g/dL	10.0 (8.2–12.4)	10.8 (9.4–13.2)	0.29
Excision, %TBSA	4.0 (2.5–6.0)	4.0 (3.5–9.0)	0.50
Harvest, %TBSA	3.0 (2.0–4.0)	3.0 (3.0–6.0)	0.42
Duration of surgery, min	241 (173–271)	361 (246–408)	0.13
Duration of surgery per excision, min/%TBSA	40 (34–173)	48 (42–103)	0.54
Duration of tourniquet, min	0	68 (55–98)	<0.001
Blood loss, g	275 (113–4617)	495 (366–2206)	0.54
Blood loss per %excision, g/%TBSA	275 (28–769)	100 (73–428)	0.93
Blood loss per %excision and %harvest, g/%TBSA	138 (16–432)	53 (44–244)	0.93
Crystalloid, mL	1800 (1325–6850)	2675 (2287–3427)	0.18
HES,^a^ mL	0 (0–1200)	500 (0–1125)	0.75
RCC-LR,^b^ units	0 (0–10)	2 (0–8)	0.78
RCC-LR per %excision (units/%TBSA)	0.0 (0.0–1.7)	0.32 (0.0–1.0)	0.74
Graft thickness (×1/1000 in)	12 (12–13)	25 (25–28)	<0.001
Donor site (number of harvests)			<0.01
Dorsum of the trunk	0	8	
Thigh	4	0	
Abdomen	2	0	

Continuous variables are expressed as median (interquartile range)

*FFP-LR* leukocyte-reduced fresh frozen plasma, *HES* hydroxyethyl starch solution, *n* number, *RCC-LR* leukocyte-reduced concentrated red blood cells

^a^HES used in our study was as follows: 6 mass/volume percent; mean molecular weight, 70,000 Da; degree of substitution 0.55

^b^One unit of RCC-LR in our study was processed from 200 mL of donated blood

### Postoperative course

Time from the operation to the start of active motion exercises was significantly shorter for the FTD group [13 (10 − 15) vs 7 (5 − 7) days; *P* < 0.01] (Table 4). The percentage of graft take of the dorsum of the hand 7 days postoperatively was significantly higher in the FTD group than that in the PTD group [90 (85 − 90) % vs 98 (95 − 99) %; *P* < 0.01]. The time required for donor site epithelialization was significantly shorter in the PTD group than that in the FTD group [9 (7 − 16) vs 25 (19 − 30); *P* < 0.01]. Twelve months after the operation, there was no significant difference between groups in the modified Vancouver Scar Scale of the donor site and in the occurrence of hypertrophic scarring and scar contracture of the grafted hand; however, the incidence of epidermal cysts was significantly higher in the PTD group than that in the FTD group (4 of 6 vs 0 of 8; *P* = 0.02).

**Table 4:** Postoperative course

	Debridement group		*P* value
	Partial-thickness (*n* = 6)^a^	Full-thickness (*n* = 8)^a^	
Time from operation to start of active motion exercise, days	13 (10–15)	7 (5–7)	<0.01
Graft take 7 days postoperatively, %	90 (85–90)	98 (95–99)	<0.01
Donor site epithelialization, days	9 (7–16)	25 (19–30)	<0.01
Modified Vancouver Scar Scale of donor site	4 (3–6)	5 (3–6)	0.75
Hypertrophic scar of hand, *n*	3	2	0.58
Contracture, *n*	1	1	1.00
Epidermal cyst, *n*	4	0	0.02

Continuous variables are expressed as median (interquartile range)

*n* number of hands

^a^Number of hands operated on

### Hand esthetic outcomes

**Fig. 7:**
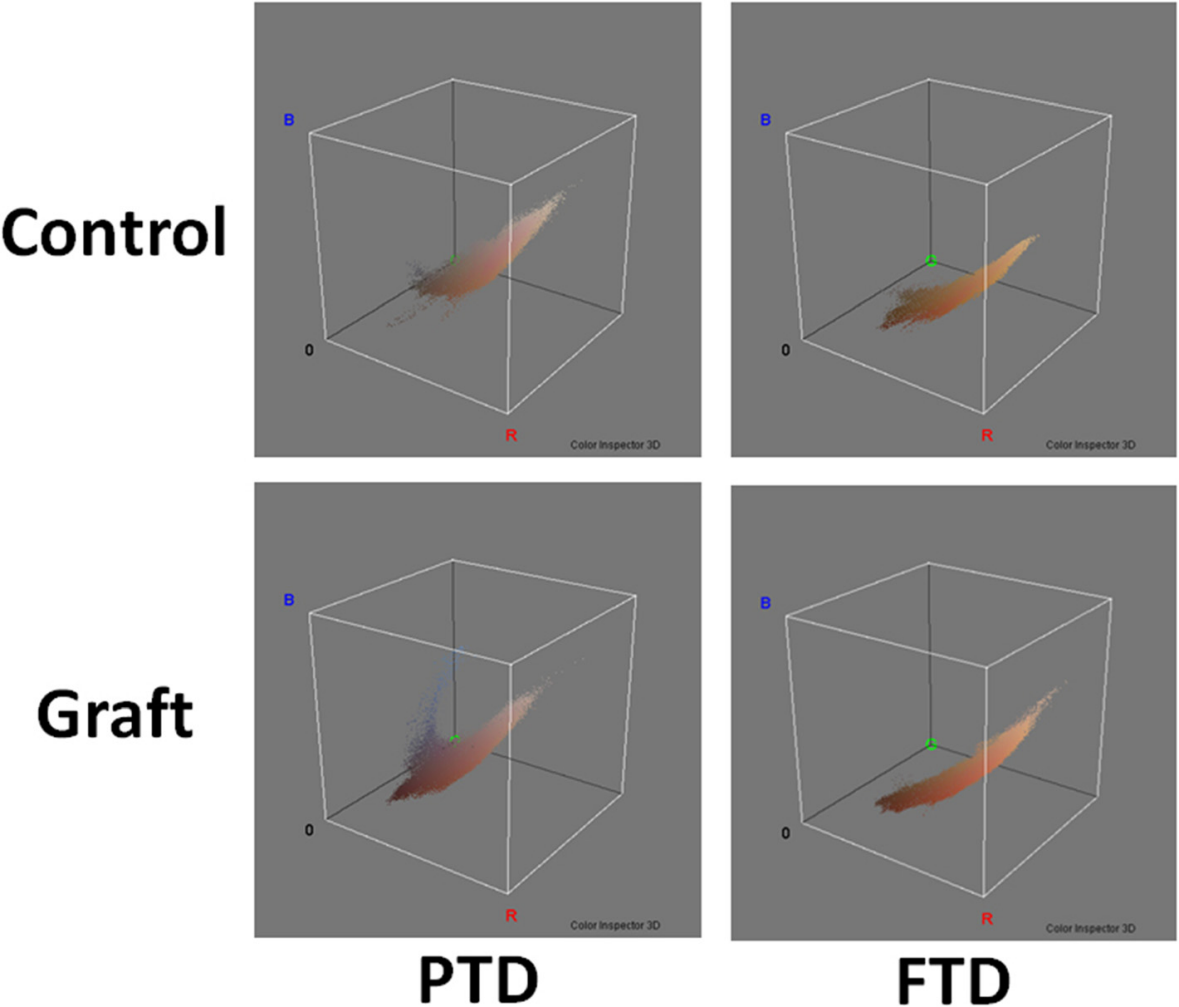
Three-dimensional histograms in the red, green, and blue (RGB) color space. Results shown for the RGB color space of the control area and the skin graft in the partial-thickness debridement (*PTD*) group and the full-thickness debridement (*FTD*) group. In the 3D histogram of the skin graft of the *PTD* group, there is a group of dots which cannot be observed in the control. In the skin graft of the *FTD* group, a group of dots that cannot be interposed on dots of the control is not obvious

**Fig. 8:**
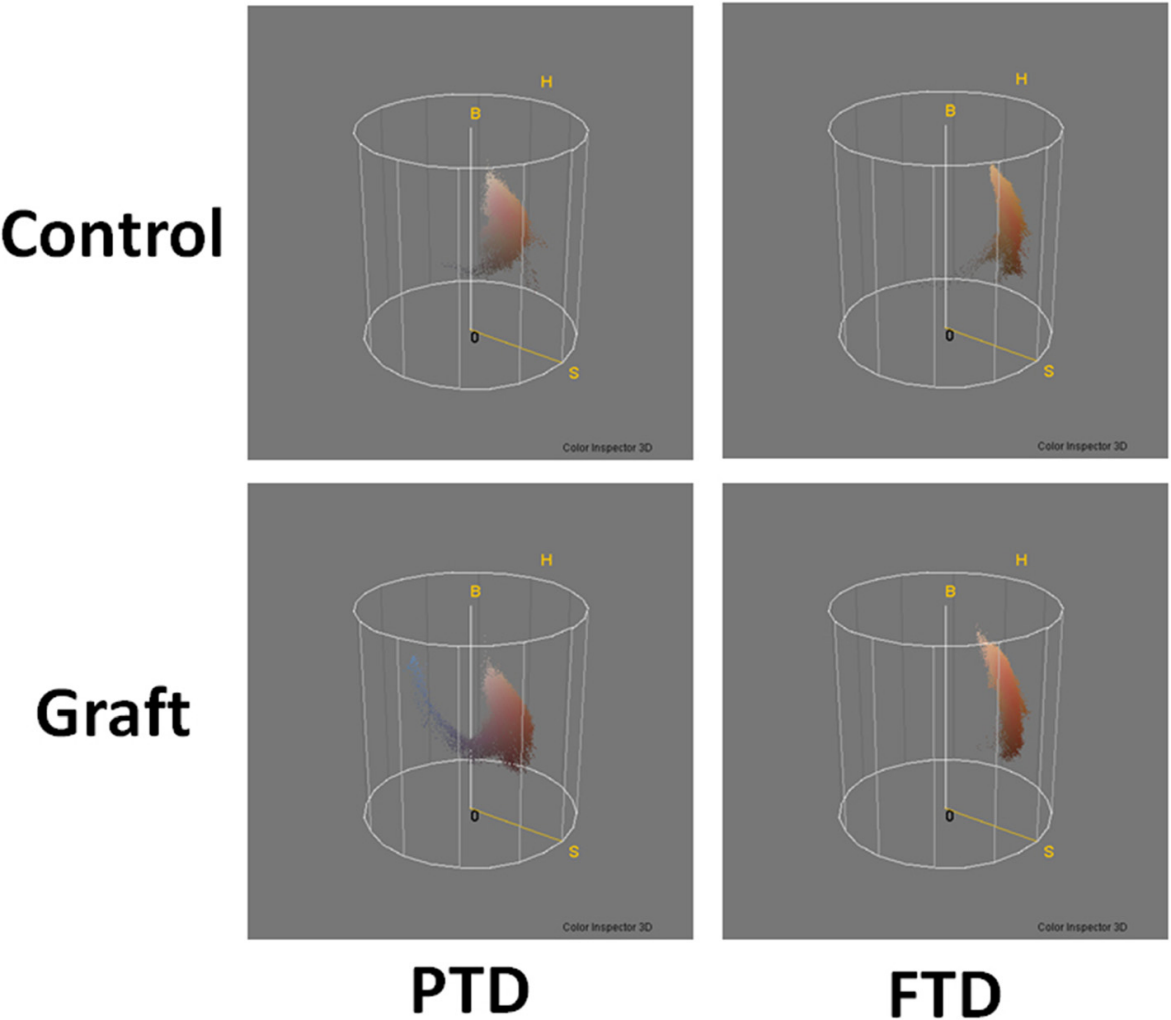
Three-dimensional(3D) histograms in the hue, saturation, and brightness (HSB) color space. The shape formed by the histogram dots was similar between the control and graft areas in the full-thickness debridement (*FTD*) group, but not similar between the control and graft areas in the partial-thickness debridement (*PTD*) group

The 3D histograms in both the RGB and HSB color space showed distribution differences between the PTD group and the FTD group (Figs. 7 and 8). In the FTD group, the shape formed by the histogram dots was similar between the control and graft areas, and the dots were grouped in one area, whereas in the PTD group, the histogram dots in the graft area consisted of one major group and a separate minor group and these were unlike the dots observed in the control area.

**Fig. 9:**
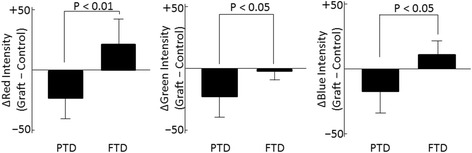
Differences in the weighted mean value of intensity of red, green, and blue (RGB) color components. The absolute values of Δred intensity, Δgreen intensity, and Δblue intensity were significantly higher in the partial-thickness debridement (*PTD*) group than those in the full-thickness debridement (*FTD*) group. Differences (graft-control) in all three color components of the RGB color space (red, green, and blue) in the PTD group were negative, indicating that the graft is darker than the control

In the 2D histograms of each RGB color component (red, green, and blue), the absolute values of Δred intensity, Δgreen intensity, and Δblue intensity were significantly higher in the PTD group than those in the FTD group, indicative of the larger differences in color between the control and the grafted skin in the PTD group (Fig. 9). All Δred intensity, Δgreen intensity, and Δblue intensity values were negative in the PTD group, which indicated that the grafted skin was darker than the control skin area in the PTD group.

**Fig. 10:**
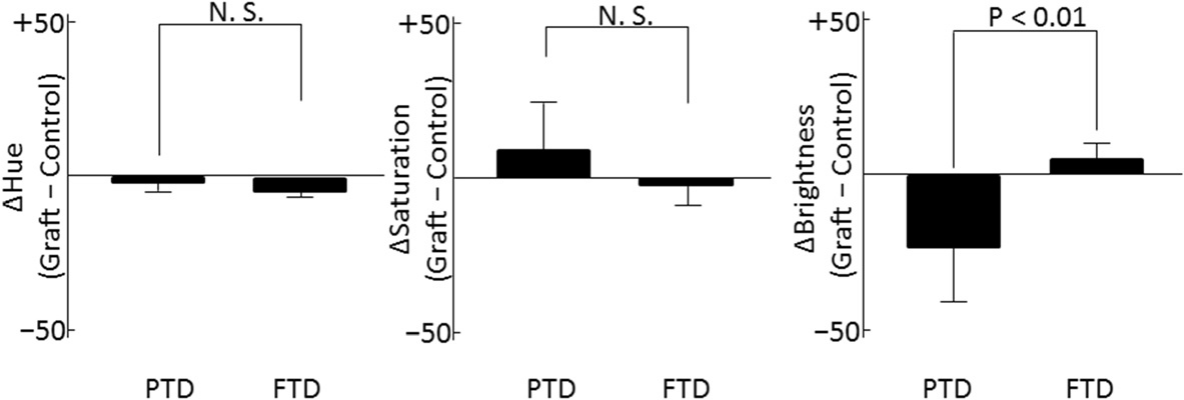
Differences in weighted mean value of each of the color components of hue, saturation, and brightness (HSB). The absolute values of Δbrightness were significantly larger in the partial-thickness debridement (*PTD*) group than those in the full-thickness debridement (*FTD*) group. Difference (graft-control) in brightness of the PTD group is negative, indicating the graft is darker than the control. *N.S.* not significant

Based on the 2D histograms of HSB color components, the Δhue and Δsaturation were not significantly different between the two groups. The absolute value for the Δbrightness in the PTD group was significantly higher than that for the FTD group, which indicated that the difference in brightness between the graft and the control tissue was significantly larger in the PTD group than that in the FTD group (Fig. 10). The Δbrightness value in the PTD group was negative, which indicated that the graft was darker than the control skin.

## Discussion

This is the first study that has directly compared the esthetic outcome of PTD followed by medium STSG and FTD followed by thick STSG as a treatment for a deep dermal burn of the dorsum of the hand. Hyperpigmentation is caused by the excessive accumulation of melanin, and hyperpigmented skin looks darker than skin with normal pigmentation [21–23]. Our study suggested that a thick graft from the dorsal trunk after FTD is an option that can reduce the risk of hyperpigmentation after surgery for burns of the dorsum of the hand in Japanese patients. In our study, graft take and the appearance of the hand were significantly better in the FTD group compared with those in the PTD group. There were no differences in function between the two groups. We considered that the reduced pigmentation in the FTD group compared with the PTD group was caused by the composite effect of sufficient debridement, dorsal trunk donor site, and the thickness of the skin graft.

Graft take was significantly better in the FTD group in our study. We suggest that this was mainly caused by sufficient debridement [13, 24]. There are fewer reports about full-thickness debridement for surgery of a deep dermal burn of the dorsum of the hand than there are for tangential excisions [1, 2, 14, 15, 25–27]. Our treatment strategy in the FTD group is fundamentally in line with a report by Gurfinkel et al. [13]. Improved graft take in the FTD group might have caused less pigmentation of the graft. We think that FTD combined with usage of a tourniquet makes it easier to remove burned tissue completely. Gurfinkel et al. reported that, in a histological analysis, 21.8 % (32 of 146) of samples of tangentially excised (i.e., partial thickness debridement) burn eschars that were clinically deemed to have reached a required depth of debridement did not reach viable tissue [13]. A newly developed water jet hydrosurgical system (Versajet®) may be a workable option to achieve sufficient PTD under a near-bloodless field without a tourniquet; however, we have had no experience with this system [20, 28–32].

In our study, other possible factors that might have influenced the pigmentation of skin grafts were the differences in skin graft donor site and graft thickness [33]. In the PTD group, the lateral thigh or abdomen was used as the donor site for the medium skin graft, whereas the dorsal trunk was used as the donor site for the thick skin graft in the FTD group.

There were significantly more days to donor site epithelialization in the FTD group in our study. A very thin STSG to the donor site following thick skin graft harvest may resolve the prolonged healing problem of the thick skin graft harvested site [34]. To avoid thick STSG harvest, a combination of debridement and collagen-elastin matrix (Matriderm®) as a dermal substitute, followed by a thin STSG, as reported by Haslik et al., can be chosen; however, we have had no experience with the method [35].

In the color space analyses of our study, the skin graft in the PTD group was significantly darker than it was in the FTD group. Analysis of the accumulation of melanin in the skin using RGB color channels, or other color channels, generated from a conventional color photographic image, has been reported by several authors [18–20, 36, 37]. People of Asian race show stronger pigmentation reactions than do Caucasians because of differences in genetic background and therefore skin physiology [4, 9–12, 38]. The strong pigmentation of medium split-thickness skin grafts observed in our study is consistent with the preceding reports [6]. Tsukada reported that, using electron microscopy, it can be seen that changes in transfer, formation, distribution, degradation pattern, and the size of melanosomes in keratinocytes cause hyperpigmentation of skin grafts in Japanese people [39].

The main limitation of this study is the small sample size, which can cause a type 2 statistical error. The retrospective nature of the study makes it difficult to infer causality. Multiple factors, such as depth of debridement, donor site, and the graft thickness, are affecting the outcome. Our study cannot differentiate the effects of each of these factors. The generalizability of our results is unknown because the study only included Japanese patients in a limited number of hospitals.

## Conclusions

FTD followed by a thick STSG from the dorsal trunk may be an option that can reduce the risk of hyperpigmentation after surgery for burns of the dorsum of the hand in Japanese patients. Further investigation is needed to clarify whether the FTD or the thick STSG or both are the factor for the control of hyperpigmentation.

## Abbreviations

%TBSA: percentage of total body surface area
2D: two-dimensional
3D: three-dimensional
DDBs: deep dermal burns
FTD: full-thickness debridement
HSB: hue, saturation, and brightness
PTD: partial-thickness debridement
RGB: red, green, and blue
STROBE: Strengthening the Reporting of Observational Studies in Epidemiology
STSG: split-thickness skin graft
